# Conservation of Tiger Nut Tubers with Natural Biofilm Formulated with *Thymus zygis* Essential Oil

**DOI:** 10.3390/molecules30030436

**Published:** 2025-01-21

**Authors:** M. Pilar Santamarina, Silvia Giménez-Santamarina, Cristina Santamarina, Silvina Larran, Josefa Roselló

**Affiliations:** 1 Departamento de Ecosistemas Agroforestales, Universitat Politècnica de València, Camino de Vera s/n, 46022 Valencia, Spainjrosello@upvnet.upv.es (J.R.); 2 Departamento Bayer Crop Science, Escuela Técnica Superior de Ingeniería Agronómica y del Medio Natural (ETSIAMN), Universitat Politècnica de València, Camino de Vera s/n, 46022 Valencia, Spain; csantama@agf.upv.es; 3 Centro de Investigaciones de Fitopatología (CIDEFI), Facultad de Ciencias Agrarias y Forestales, Universidad Nacional de La Plata, La Plata 1900, Argentina

**Keywords:** *Cyperus esculentus*, chufa, tiger nut, *Thymus zygis*, essential oil, preservation, antifungal effect

## Abstract

*Cyperus esculentus* L. var *sativus* is cultivated in Spain, only in the L’Horta Nord in the Valencia region. In this country, tubers are consumed fresh to make a popular beverage in the Valencia region called “horchata de chufa” (chufa milk). This drink is considered beneficial for human health thanks to its high nutritional value and medicinal importance in several treatments. This work evaluates the antifungal potential of the *Thymus zygis* essential oil against fungi found in tiger nut warehouses to preserve tubers under the best conditions. The analyzed commercial thyme essential oil belongs to the thymol/p-cymene/γ-terpinene chemotype. Thymol was found in larger quantities (51.34%), followed by the identified biogenetic precursors p-cymene (35.16%) and γ-terpinene (3.53%). Carvacrol also appeared, but in small quantities (3.53%). During in vitro tests, the *T. zygis* EO showed strong inhibition (98.85% to 91.81% MGI) against fungi *Alternaria alternata*, *Fusarium andiyazi*, *Fusarium incarnatum*, and *Fusarium oxysporum* at 300 µg/mL. It totally inhibited their growth (100% MGI) at 400 µg/mL, and did so strongly (75.94%, 72.02%, and 70.78%) with fungi *Podospora australis*, *Penicillium commune*, and *Cladosporium subuliforme*, respectively. Under in vivo conditions, formulated as a protective biofilm, and by forcing the environmental conditions of temperature and humidity to the maximum for fungus *F. andiyazi* growth on tiger nut tubers, the created film acted as a strong protector against fungal attacks.

## 1. Introduction

*Cyperus esculentus* L. belongs to the genus *Cyperus* within the *Cyperaceae* family, which due to its diversity of species is the third family of Monocots. *Cyperus* is the largest genus of the *Cyperaceae*, with about 700 species [1]. The Latin name *Cyperus* derives from the Greek *kýpeiros*, which refers to a kind of rush, while *esculentus* means “edible”, in relation to its underground tubers [2]. This species has been known to be edible for more than 3000 years, with tubers found in Tutankhamen’s tomb [3].

*C. esculentus* has great plasticity and is widespread in tropical, subtropical, and temperate regions worldwide [4,5,6]. It also possesses vast variability, thanks to which several authors have focused on studying the infraspecific taxonomy of *C. esculentus*. This was started by Boeckeler [7] for the American forms and cultivated varieties (var. *sativus* Boeckeler). Clarke [8] and Britton [9] have described new varieties from North America and India, which is interesting. Before reviewing taxa, in 1936, Kükenthal [10] proposed further *C. esculentus* varieties, and recently, others have been registered to show the species’ wide variability [11].

*C. esculentus*, known as chufa, juncia avellanada, tiger nut, yellow sedge, and earth almond, among others, includes wild and selected varieties for cultivation [12]. Varieties that grow wild in some regions are considered one of the worst weeds of agricultural and horticultural crops in the world, because of their invasive growth due to their root system, which is difficult to control. Interestingly, *C. esculentus* var. *sativus* includes the species selected by humans for cultivation, which have larger and sweeter tubers than wild varieties [13]. Tiger nut is a perennial herb that produces rhizomes, stolons, and terminal geophyte tubers, although cultivated varieties often display annual behavior. Tuber size can lie between 15 mm and 25 mm in diameter in wild and cultivated varieties, respectively [2,4].

There are pieces of evidence that prove that ancient Egyptians planted and used this *Cyperus* for culinary and medicinal purposes. Nowadays, the use of this *Cyperus* for food and medicinal purposes is widely known because it is rich in fat, protein, sugar, and other nutrients like volatile oil, organic acids, alkaloids, phenols, terpenoids, anthraquinone, and steroids, etc., with diverse properties for human health [5]. Its tuber composition has recently been analyzed to show a high calorie content with a high percentage of starch (more than peanut and soybean), high contents of lipids (more than soybean), carbohydrates (more than peanut and sugar), dietary fiber, proteins, alkaloids, vitamins, and minerals, among others [5]. Likewise, other authors have recorded the presence of different substances, such as flavonoids, terpenoids, and tannin, along with other biological and pharmaceutical uses [14].

*C. esculentus* is distributed worldwide in tropical, subtropical, and temperate regions. The *sativus* variety is cultivated in some regions of Africa, where it is consumed fresh, dried as a snack, and also used as an alternative to flour [15]. In America (Chile, Brazil, and the United States), this crop is used as animal feed. In Asia (China and India) and Europe, its uses differ. In China, tiger nut is predominantly cultivated as a strategic alternative to the scarcity of oil crop resources, which are of high nutritional quality [15]. In Spain, the crop is cultivated only in the L’Horta Nord of the Valencia region [4]. Annually, this region produces around 5.3 million kilograms of dry chufa, of which 90% has had Protected Designation of Origin since 1995 as “Chufa de Valencia/Xufa de València” by the Conselleria d’Agricultura, Medi Ambient, Canvi Climàtic I Desenvolupament Rural [16]. In Spain, tubers are consumed fresh to make a popular beverage in the Valencia region called “horchata de chufa” (chufa milk). Interestingly, this drink is considered beneficial for human health because of its high nutritional value and its medicinal importance in several treatments [17]. Nowadays, different products are made based on chufas, such as chocolates, beer, liquor, and gin, and even in the cosmetic industry [4,18].

There is evidence that different genera of fungi, such as *Ascochyta*, *Cercospora*, *Cintractia*, *Claviceps*, *Dactylaria*, *Fusarium*, *Puccinia*, and *Sclerotinia*, are associated with *C. esculentus* plants growing as weeds, and most have been found outside Europe [4]. For example, *Sclerotinia minor* has been cited to cause symptoms in yellow sedge plants growing in peanut fields in the United States [19]; rust *Puccinia canaliculate* is registered in different countries [20]; and *Cercospora caricis* is reported to cause foliar disease in North Carolina (USA) [21], among others.

However, very little attention has been paid to the phytosanitary problems of the crop of *C. esculentus* var. *sativus*. “Tuber rot” caused by *Dematophora necatrix* Hartig (teleomorph: *Rosellinia necatrix* Prill.) was registered in 1997 in the Valencia Province of Spain [22,23].

In 2022, a virus that caused reduced plant growth, chlorosis, and mosaic in leaves and root atrophy that drastically reduced tuber size was identified as Xufa yellow dwarf virus (XYDV) [24]. Marsal et al. [25] have reported a disease that causes external black spots on tubers, known as “black chufa” or “black spot”; however, the etiology of this pathology is still unknown.

It is interesting to know that once the crop cycle finishes, plants are uprooted, and the tubers are collected, washed, and selected to remove defective tubers. Cleaned chufas are left inside “drying attics” or “cambras” to reduce humidity from 50% to 11%. This is because high moisture content can cause tuber rancidity and fungal growth, which consequently reduces their quality and commercial value. Therefore, the drying process period is considered extremely important after harvest. During this period, which usually lasts about 3 months, chufa tubers develop their natural sweetness; however, they can still be affected by different phytosanitary problems.

It is widely known that fruit and vegetable fungal infection may occur on field crops and along the entire food chain, including harvesting, transport, packing management, postharvest storage, and marketing. This contamination by fungi along the food chain impacts fruit and vegetables, and can cause 35–55% reductions in yield and market quality [26,27]. Spoilage fungi invade foods and cause quality reduction, which render products unesthetic or unusable, and sometimes unsafe. Some fungi also produce mycotoxins as secondary metabolites, which are dangerous to human health [28,29].

The tiger nut crop has been paid little attention in terms of production and genetic improvement and therefore, yields are low and susceptible to diseases and pests. No reports have been found about fungi isolated from tiger nuts during storage, which can cause yield losses, including deterioration of quality and a shorter shelf life, and may also produce mycotoxins, which are capable of harming human health.

In this work, we focused on studying the tuber-borne fungi and the fungi present in drying chambers. Once fungi were isolated, we focused on evaluating an ecofriendly alternative against these fungi to preserve shelf life and chufa tuber quality during the postharvest.

Different innovative alternatives to synthetic additives to reduce the risk of food contamination and preserve the shelf life of food have been proposed with promising results. Among them, there are reports of nanomaterials and essential oils used in food packaging to prevent food spoilage, and to prolong food shelf life [30,31]. Essential oils (EOs) are known for their applications in foods, in the pharmaceutical industry, and for their wide use as antimicrobials, mainly due to their bioactive compounds. Several authors have recorded the potential effect of EOs to protect and preserve foods against pathogenic and spoilage microorganisms [32,33].

The genus *Thymus* has a wide chemical polymorphism with several chemotypes. Interestingly, a significant amount of research has demonstrated antibacterial and antifungal activity [34].

Thus, the objectives of the present work were as follows: (i) isolate and identify the fungal population from chufa tubers and airborne fungal cultivable species from drying chambers; (ii) evaluate the antifungal potential of the *Thymus zygis* EO against isolated fungi in an in vitro assay; and (iii) assess the effectiveness of the *T. zygis* EO applied as a natural biofilm for tiger nut preservation purposes.

## 2. Results

### 2.1. Chemical Composition of the Thymus zygis EO

In the commercial thyme analyzed herein (Table 1), chemotype thymol/p-cymene/γ-terpinene was found in the largest quantities of thymol (51.34%), followed by the identified biogenetic precursors p-cymene (35.16%) and γ-terpinene (3.53%). The other identified compounds included oxygenated monoterpenes carvacrol (3.53%), linalool (2.21%), and borneol (1.12%), with percentages above 1%. Only two sesquiterpene hydrocarbons were detected: β-caryophyllene (0.13%) and α-humulene (0.02%).

### 2.2. In Vitro Studies. Determining the Antifungal Potential of the Thymus zygis EO. MGI (%) (Mycelial Growth Inhibition)

**Figure 1:**
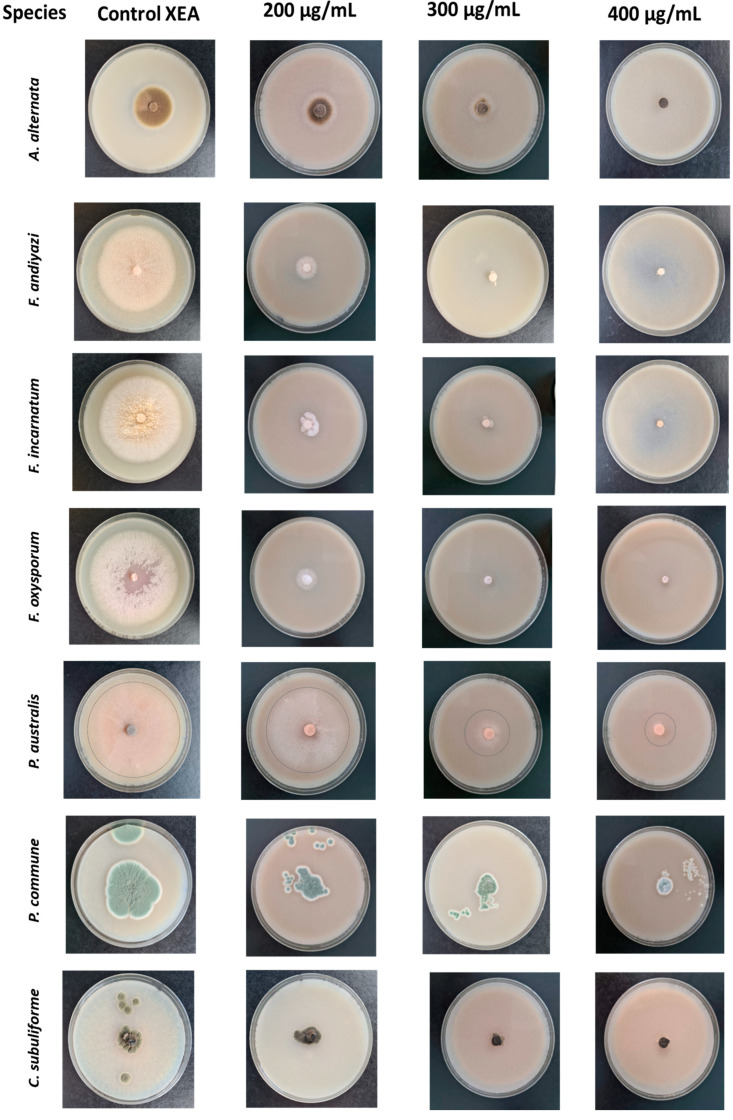
Mycelial growth of seventh day of *Alternaria alternata*, *Fusarium andiyazi*, *Fusarium incarnatum*, *Fusarium oxysporum*, *Podospora australis*, *Penicillium commune*, and *Cladosporium subuliforme* on Control (XEA) at different concentrations (200, 300, and 400 μg/mL) of *Thymus zygis* essential oil.

The results obtained in this assay showed that the *Thymus zygis* EO reduced the fungal growth of all the evaluated phytopathogens (Table 2 and Table 3, Figure 1). EO inhibition was affected by the doses used in all the tested fungi. The MGI increased the higher the doses became. *Fusarium andiyazi* and *Fusarium incarnatum* were the most sensitive fungi at the 200 µg/mL dose.

The *T. zygis* EO showed strong inhibition (98.85% to 91.81% MGI) against *Alternaria alternata*, *F. andiyazi*, *F. incarnatum*, and *F. oxysporum* at 300 µg/mL. It totally inhibited the growth of these fungi (100% MGI) at 400 µg/mL, and strongly inhibited it with 75.94%, 72.02%, and 70.78% for fungi *Podospora australis*, *Penicillium commune*, and *Cladosporium subuliforme*, respectively.

Fungi *P. australis*, *P. commune*, and *C. subuliforme* showed minor, albeit not negligible inhibition, with MGI values of 41–52% at doses 200–300 µg/mL, except for *C. subuliforme*, whose inhibition was very low at 200 µg/mL.

**Figure 2:**
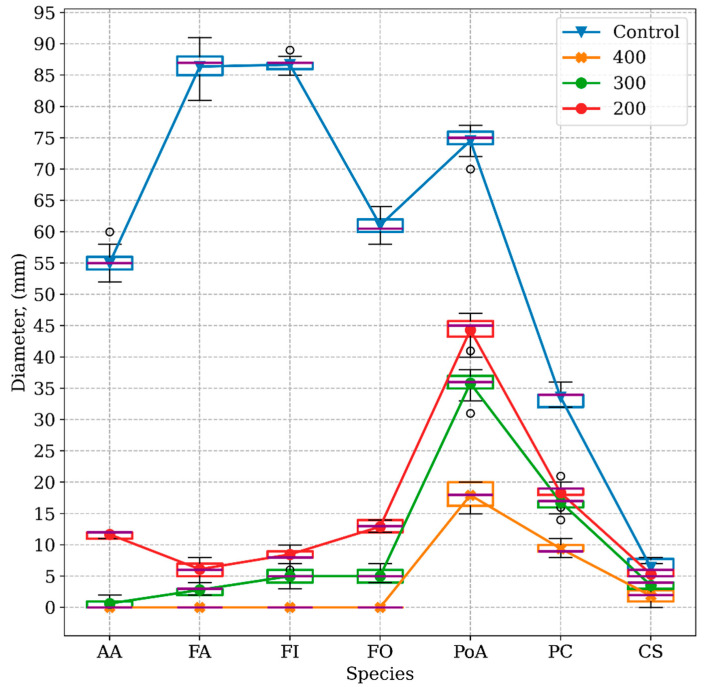
Interaction plot, mean growth, species, at 200, 300, and 400 μg/mL concentrations of *Thymus zygis* essential oil against *Alternaria alternata* (AA), *Fusarium andiyazi* (FA), *Fusarium incarnatum* (FI), *Fusarium oxysporum* (FO), *Podospora australis* (PoA), *Penicillium commune* (PC), and *Cladosporium subuliforme* (CS). *n* (30) observations per treatment were used in the statistical analysis.

The inhibitory effect of the *Thymus zygis* EO on the growth of seven fungal species at 200, 300, and 400 µg/mL was evaluated by Tukey’s HSD plots. The results showed a significant MGI for all the species tested at three doses compared to the control (*p* ≤ 0.05) (Figure 2). This inhibition was greater at the 400 µg/mL dose, as the graphs depict.

Figure 2 shows the marked effectiveness of all the tested doses of *T. zygis* essential oil on the tested fungi. Significant differences were found in the mean growth of all fungi under all the tested conditions when the EO was used at the different doses compared to the mean growth of the controls. One exception was *Alternaria alternata*, in which case the 300 and 400 µg/mL doses were equally effective.

Finally, the results showed that, when comparing the inhibitory effects of the three EO doses tested, at the 400 µg/mL concentration, the *Thymus zygis* extract completely inhibited (100%) the growth of *Alternaria alternata*, *Fusarium andiyazi*, *Fusarium incarnatum*, and *Fusarium oxysporum*.

Therefore, the *Thymus zygis* EO at 800 µg/mL was selected to study its effect on harvested and stored tiger nut conservation to prolong their commercial shelf life.

### 2.3. In Vivo Study of the Antifungal Effect of the Thymus zygis EO Against Fusarium andiyazi on Tiger Nut Storage

**Figure 3:**
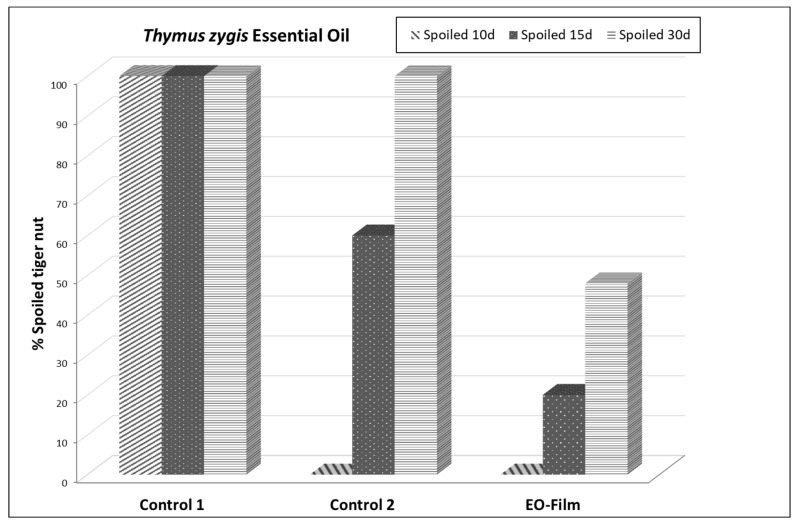
Effect of *Thymus zygis* essential oil at 800 µg/mL on *Fusarium andiyazi* inoculated in tuber tiger nuts after 10, 15, and 30 days. Control 1, fruits without film and without EO. Control 2, fruits with film and without EO. EO-Film, fruits with film and essential oil.

In this study (Table 4, Figure 3), the protective effect of the film with the EO was observed, because it maintained tuber turgor, prevented tuber weight loss, allowed *Fusarium andiyazi* infection to not advance, and functioned as a second epidermis.

Two days after the experiment began, all the tubers were healthy in both the control and treatments. After 10 days at 90% RH, all the tubers were damaged in the control. In control 2 (covered with film without the EO), 68% of tubers were healthy, which indicates a certain protective effect of film. Those covered with film containing the *Thymus zygis* EO (EO-film) were completely healthy (96%), or only the inoculation point was evident (4%) with no damaged tubers. On the 15th day of the experiment, 60% of the tubers in control 2 were damaged, 40% had a stained inoculation point and none were healthy. Of those covered with the film containing the *T. zygis* EO (EO-film), 84% were healthy or had a patent inoculation point.

At 30 days, of all the tubers in both control 1 and control 2, 100% were damaged. Of those treated with the film containing the EO, 52% were healthy or had a somewhat patent inoculation point. From this, we can conclude that the film containing the EO offers a good protective effect, and the film without the EO has a certain protective effect.

## 3. Discussion

This study is the first to report on the mycobiota of tiger nut tubers collected from the field and stored. We found that the *Fusarium* genus predominated with different species, of which some produced mycotoxins. We also found the cosmopolitan fungus *Alternaria alternata*, some *Penicillium* species, a typical storage fungal genus, and some Mucoral. No species of the *Aspergillus* genus were detected, nor fungus *A. flavus*.

As is known, the presence of mycotoxins in feeds and foods can cause different adverse health effects and pose a serious health threat to both humans and livestock. In previous studies, the presence of mycotoxins such as deoxynivalenol (DON), aflatoxins, and ochratoxin A and BEA has been demonstrated in tiger nuts obtained from different local markets and cooperatives from the Valencian Community (Spain [36]). *Aspergillus*, *Fusarium*, and *Penicillium* are the three fungal genera that dominate mycotoxin production, are consistently detected in food and feed, and can appear in the food chain as a result of fungal infection of crops and after harvest [37]. Interestingly, we obtained fungal species from two of these genera from tiger nut tubers collected from the field and stored.

According to the results obtained in our research, tiger nuts are a favorable substrate for the development of fungi that could potentially produce mycotoxins. Therefore, we highlight our promising results obtained with *T. zygis* essential oil on the reduction in fungal species found in tiger nut tubers.

The genus *Thymus* is highly polymorphic, and its species exhibit distinct chemical profiles. A previous study conducted on *Thymus serpyllum* and two *T. piperella* chemotypes observed that the analyzed *T. serpyllum* species was rich in thymol and carvacrol, in a very balanced proportion (21.5% and 18.7%, respectively). The two *T. piperella* chemotypes exhibited different chemical profiles. *T. piperella* chemotype 1 was rich in carvacrol (51%), while *T. piperella* chemotype 2 was rich in thymol (35.7%). The study of the antifungal activity of these EOs at the 300 µg/mL dose against the phytopathogenic and postharvest fungi *Alternaria alternata*, *Bipolaris spicifera*, *Curvularia hawaiiensis*, *Fusarium oxysporum*, *Penicillium italicum*, and *Botryotinia fuckeliana* revealed that the species containing the mixture of both monoterpenes had a higher antifungal potential. In other words, both compounds play a synergistic role by enhancing their antifungal effect [33]. The same synergism results were obtained in the study of different botanical compounds when tested independently and when their mixtures were tested against fungi *Botryotinia fuckeliana* and *Rhizoctonia solani*. The mixture of the botanical active ingredients had a synergistic effect and gave better results [38].

In our study, the major components of the commercial *T. zygis* oil were thymol (51.34%), followed by the identified biogenetic precursors p-cymene (35.16%) and γ-terpinene (3.53%), and a smaller amount of carvacrol. This means that they correspond to chemotype thymol/p-cymene/γ-terpinene. The synergism shown by the components of the *T. zygis* EO makes it especially effective against the tested fungi, with growth inhibition over 70% against them all, and total growth inhibition in four of the seven fungi studied in the in vitro test. Furthermore, under the in vivo conditions, formulated as a protective biofilm and forcing the environmental temperature and humidity conditions to the maximum for fungus *F. andiyazi* to grow on tiger nut tubers, after 15 days, 84% of tubers were ready for consumption. These conditions do not occur in warehouses because they have drying nozzles and temperature and humidity controls. The tiger nuts harvested in summer are stored for horchata production, and the conditions of autumn in Valencia are hot and humid, with cold snaps and cold drop episodes, hence the importance of keeping tubers under the best conditions to be able to produce the quality drink D.O. horchata of Valencia.

Studies have pointed to the antifungal potential of thyme (*Thymus vulgaris* L.) essential oil, which contains p-cymene (36.5%), thymol (33.0%), and 1,8-cineole (11.3%) as main components, against fungal species from damp dwellings [39]. Additionally, various studies have shown the very high potential of the EO in the control of phytopathogenic and postharvest fungi, the importance of its formulation in biofilm formation, and its protective role in fruit, vegetables, seeds, and cereals (tomatoes, persimmons, and rice, etc.) by extending the shelf life of treated products [26,32,33]. In addition, Sapper et al. [40,41] published works in 2018 and 2019 that studied the physico-mechanical properties and the antifungal activity of a created biofilm containing the *Thymus zygis* EO, with excellent results. This shows the very high potential of this EO against pathogenic and spoilage microorganisms.

## 4. Materials and Methods

### 4.1. Fungal Species

The fungal species employed in this study were as follows: *Alternaria alternata* (Fr.) Keissler (LBEA 2300), *Fusarium andiyazi* Marasas, Rheeder, Lampr., K. A. Zeller and J. F. Leslie (LBEA 2303), *Fusarium incarnatum* (Desm.) Sacc. (LBEA 2304), *Fusarium oxysporum* Schltdl. (LBEA 2305), *Podospora australis* (Speg.) Niessl (LBEA 2302), *Penicillium commune* (LBEA 2307), and *Cladosporium subuliforme* Bensch, Crous and U. Braun (LBEA 2310). They were all isolated from Alboraya (Valencia) tiger nut tubers in the Laboratorio Botánica of the Departament of Ecosistemas Agroforestales (LBEA), Escuela Técnica Superior de Ingeniería Agronómica y del Medio Natural (ETSIANM), and Universitat Politècnica de València (València, Spain). All the isolated fungi were maintained on PDA (Potato Dextrose Agar) and on XEA (Chufa Extract Agar) at 5 °C until they were used.

### 4.2. Fungal Strains Identification

These fungi were identified by morphological and molecular methods. The morphological analysis consisted of the inoculation, incubation, and validation of culture characteristics, as well as microscopic observation, growing data, or colony morphology. The molecular identification of fungal species was carried out by the Laboratorio de Técnicas Instrumentales of the Universidad de León, Spain. Two different regions of ribosomal DNA genes were analyzed: the nuclear ribosomal internal transcribed spacer ‘ITS region’, including 5.8S rDNA as a standard barcode for fungi [42]; and the D1/D2 hypervariable domains of large subunit 28S rDNA (LSU). The analysis of sequences was performed by BLAST similarity for the ITS region and the D1/D2 domains of the LSU gene against the public database RefSeq: NCBI Reference Sequence DataBase (URL http://www.ncbi.nlm.nih.gov/refseq/ accessed on 18 July 2018).

### 4.3. Essential Oil (EO)

The commercial *Thymus zygis* EO (batch 105, date January 2026), prepared from leaves, stems, and flowers, was purchased at Plantis, Artesanía Agricola S.A. (Ctra. a Vilafranca, C-15 B, Km 5,2, 08810 Sant Pere de Ribes Barcelona, Spain). EOs were stored at 4 °C until the chemical analysis and antifungal studies were conducted.

### 4.4. Gas Chromatography/Mass Spectrometry Analysis of EOs

The analysis of EOs was carried out by gas chromatography coupled to flame ionization detection (GC-FID) and mass spectrometry (GC-MS), as described in Giménez-Santamarina et al. [43]. Each sample was run in triplicate.

### 4.5. In Vitro Antifungal Activity of the T. zygis EO

An in vitro assay was performed to evaluate the antifungal activity of the *T. zygis* EO against all the studied fungi. The EO was dissolved, mixed, and homogenized by shaking in flasks in a sterilized XEA medium (500 mL chufa extract XE (60 g chufa, 1000 mL water), 16 g agar, and 500 mL water). Tween 20 (0.1%) was added at 45–50 °C to obtain final concentrations of 200, 300, and 400 μg/mL. Then, while the medium was still liquid, it was distributed on Petri dishes in a laminar flow chamber. Disks of each fungus (8 mm diameter) were taken from the edge of the 7-day colonies that developed on XEA and placed in the center of Petri dishes. Controls were performed by placing disks of each fungus on the XEA medium, but without the *T. zygis* EO.

Five replicates were performed for each fungus and *T. zygis* EO concentration. The experiment was replicated 3 times. Petri plates were incubated in the dark at 25 °C for 7 days. Measurements of the fungal colonies’ diameter were taken in two perpendicular directions and Mycelial Growth Inhibition (MGI) was calculated with the formula used by Albuquerque et al. [44]:

MGI = [(DC − DO)/DC] × 100

where DC is the average of the colonies on the control dishes and DO is the average of the colonies’ diameter of each fungus and concentration.

### 4.6. Fungitoxicity of the T. zygis EO in an In Vivo Assay

After obtaining the results of in vitro assay, an in vivo experiment was performed to evaluate the effect of the *T. zygis* EO against *F. andiyazi* (FA), which showed 100% MGI.

#### 4.6.1. Fungal Suspensions (FI) and Fungal Biofilm Preparation (FIFi)

To coat fruit with fungi, a solution containing AA and FA propagules was prepared. To do this, 10 mL of a suspension of 1 × 10^6^ conidia/mL was added to 90 mL of water/Tween 20 (0.1%) (FI). To prepare FIFi, 0.25% agar was added to the FI spore suspension. Finally, suspensions were homogenized by shaking at 170 rpm for 10 min.

#### 4.6.2. *T. zygis* EO Biofilm (EOFi)

The *T. zygis* EO concentration used to evaluate in vivo was 800 µg/mL. The *T. zygis* EO solution for coating was prepared in flasks containing water/Tween 20 (0.1%)/0.25% agar, which were homogenized by shaking at 170 rpm for 10 min.

#### 4.6.3. Biofilm Application of the *T. zygis* EO on (Tiger Nut) Tubers

A sample of chufa (tiger nut) tubers, origin Alboraya (Valencia, Spain), were surface sterilized with 1% sodium hypochlorite solution for 2 min and then washed twice with sterile distilled water for 4 min. Sterilized tubers were wounded (1 mm depth) on the surface with a sterile needle. Fifty tubers were immersed in the biofilm containing the *T. zygis* EO (EOFi) for 4 min before being placed on racks and dried for 24 h at room temperature. Afterward, tubers were immersed in FIFi for 4 min, placed on racks, and dried at room temperature for 24 h.

Two controls were performed during the trial, which consisted of the following: control 1 with 50 sterilized and wounded tubers, which were only treated with the fungal inoculum (FI) of each fungus; control 2 with 50 sterilized and wounded tubers, which were immersed in the biofilm without the EO (water/Tween 20 (0.1%)/0.25% agar) for 4 min. After 24 h, they were immersed in the fungal biofilm (FIFi) of each fungus for 4 min.

The tubers treated with the *T. zygis* EO biofilm, and controls 1 and 2, were placed on racks inside a controlled environment chamber at 90% RH and 28 °C. Tuber progress was monitored daily, and the evaluation consisted of determining incidence or severity at 10, 15, and 30 days.

### 4.7. Statistical Analysis

The effect of the *T. zygis* EO against seven fungal species was evaluated by an analysis of variance (ANOVA) for a completely randomized design using the Python v3.9, Scipy Stats module v1.15.1 (scipy.stats). Means were compared by the HSD Tukey test (*p* ≤ 0.05) available in the same Python package.

## 5. Conclusions

The *Thymus zygis* EO has a very high antifungal potential against the tested fungi: *Alternaria alternata*, *Fusarium andiyazi*, *Fusarium incarnatum*, *Fusarium oxysporum*, *Podospora australis*, *Penicillium commune*, and *Cladosporium subuliforme*. These fungi constitute the dominant mycobiota of the analyzed tiger nut tubers after harvesting and storage. The *T. zygis* EO formulated as a biofilm is highly effective and easily biodegradable. Active compounds are volatile, and the agar–agar base matrix is used in food as a gelling agent. Therefore, it does not pose any danger to human health, animal health, or the environment.

## Figures and Tables

**Table 1:** Identified compounds in commercial *Thymus zygis* essential oil.

	RT	LRI lit.	Peak Area (%) Mean ± SD	Identification Method
Monoterpene hydrocarbons			39.74 ± 0.19	
α-thujene	930	930	0.01 ± 0.00	MS, LRI
α-pinene	937	939	0.38 ± 0.01	MS, LRI
Camphene	951	954	0.02 ± 0.00	MS, LRI
*trans*-pinane	971	975	0.01 ± 0.00	MS, LRI
3-*p*-menthene	985	987	0.12 ± 0.00	MS, LRI
Myrcene	992	990	0.46 ± 0.00	MS, LRI
α-terpinene	1018	1017	0.02 ± 0.00	MS, LRI
*p*-cymene	1024	1024	35.16 ± 0.16	MS, LRI
γ−terpinene	1061	1059	3.53 ± 0.02	MS, LRI
Terpinolene	1088	1088	0.01 ± 0.00	MS, LRI
*p*-cymenene	1089	1091	0.02 ± 0.00	MS, LRI
Oxygenated monoterpenes			59.73 ± 0.34	
1,8-cineole	1033	1031	0.79 ± 0.01	MS, LRI
*cis*-linalool oxide	1074	1072	0.02 ± 0.00	MS, LRI
*trans*-linalool oxide	1086	1086	0.01 ± 0.00	MS, LRI
6,7-epoxymyrcene	1094	1092	0.01 ± 0.01	MS, LRI
Linalool	1101	1096	2.21 ± 0.03	MS, LRI
α-fenchol	1112	1116	0.01 ± 0.00	MS, LRI
Isoborneol	1156	1160	0.25 ± 0.03	MS, LRI
Borneol	1166	1169	1.12 ± 0.01	MS, LRI
terpinen-4-ol	1177	1177	0.02 ± 0.00	MS, LRI
Isocitral	1179	1180	0.05 ± 0.01	MS, LRI
Isomenthol	1180	1182	0.01 ± 0.01	MS, LRI
α-terpineol	1189	1188	0.18 ± 0.02	MS, LRI
γ-terpineol	1201	1199	0.16 ± 0.00	MS, LRI
carvacrol methyl ether	1245	1244	0.02 ± 0.00	MS, LRI
Thymol	1295	1290	51.34 ± 0.21	MS, LRI
Carvacrol	1305	1299	3.53 ± 0.01	MS, LRI
Sesquiterpene hydrocarbons			0.15 ± 0.00	
β-caryophyllene	1417	1419	0.13 ± 0.00	MS, LRI
α-humulene	1451	1454	0.02 ± 0.00	MS, LRI
Oxygenated sesquiterpenes			0.33 ± 0.00	
caryophyllene oxide	1578	1583	0.31 ± 0.00	MS, LRI
humulene epoxide II	1603	1608	0.02 ± 0.00	MS, LRI
Total identified			99.95 ± 0.53	

Compounds listed in order of elution in the column. RT: retention time according to CG/MS analysis. LRI lit.: Linear retention indices from Adams [35]. MS: Comparing with spectra from NIST 2.0. LRI: Linear retention indices based on C8-C25 alkanes. Peak area values are means ± standard deviation of three samples.

**Table 2:** Mean growth (mm) for each fungus grown on XEA (control) and XEA-*Thymus zygis* essential oil at different concentrations. XEA (Tiger nut extract agar), AA (*Alternaria alternata*), FA (*Fusarium andiyazi*), FI (*Fusarium incarnatum*), FO (*Fusarium oxysporum*), PoA (*Podospora australis*), PC (*Penicillium commune*), and CS (*Cladosporium subuliforme*).

Concentration (µg/mL)	AA	FA	FI	FO	PoA	PC	CS
Control (XEA)	54.97 ± 2.04	86.50 ± 2.80	86.67 ± 1.09	61.03 ± 1.96	74.53 ± 1.74	33.60 ± 1.30	6.40 ± 1.25
200	11.67 ± 0.48	6.10 ± 1.12	8.47 ± 1.01	12.90 ± 0.92	44.30 ± 1.88	18.30 ± 1.73	5.33 ± 0.84
300	0.63 ± 0.81	2.80 ± 0.81	5.00 ± 0.98	5.00 ± 0.98	35.80 ± 2.23	16.83 ± 0.87	3.53 ± 0.82
400	0.00 ± 0.00	0.00 ± 0.00	0.00 ± 0.00	0.00 ± 0.00	17.93 ± 1.72	9.40 ± 0.72	1.87 ± 1.04

**Table 3:** Mycelial Growth Inhibition (MGI) percentage for each fungus grown on XEA-*Thymus zygis* essential oil at different doses. AA (*Alternaria alternata*), FA (*Fusarium andiyazi*), FI (*Fusarium incarnatum*), FO (*Fusarium oxysporum*), PoA (*Podospora australis*), PC (*Penicillium commune*), and CS (*Cladosporium subuliforme*).

Concentration (µg/mL)	AA	FA	FI	FO	PoA	PC	CS
200	78.77	92.95	90.23	78.86	40.56	45.54	16.72
300	98.85	96.76	94.23	91.81	51.97	49.91	44.84
400	100	100	100	100	75.94	72.02	70.78

**Table 4:** Efficacy of the treatment with *Thymus zygis* essential oil biofilm at 800 µg/mL against fungal development of *Fusarium andiyazi* on tiger nuts.

Efficacy on Tiger Nut (%)									
	10 Days			15 Days			30 Days		
Treatment	Healthy	Spotted Spot	Spoiled	Healthy	Spotted Spot	Spoiled	Healthy	Spotted Spot	Spoiled
Control 1	0 c *	0 c	100 a	-	-	-	-	-	-
Control 2	68 b	32 a	0 b	0 b	40 a	60 a	-	0 b	100 a
EO-Film	96 a	4 b	0 b	64 a	16 b	20 b	40 a	12 a	48 b

Control 1, fruits without film and without EO. Control 2, fruits with film and without EO. EO-Film, fruits with film and essential oil. -: No Reading. * Different letters in the same column indicate a significant difference at 95% level probability by Tukey’s HSD.

## Data Availability

The raw data supporting the conclusions of this article will be made available by the authors, without undue reservation.
